# Testing for measurement invariance and latent mean differences across methods: interesting incremental information from multitrait-multimethod studies

**DOI:** 10.3389/fpsyg.2014.01216

**Published:** 2014-10-30

**Authors:** Christian Geiser, G. Leonard Burns, Mateu Servera

**Affiliations:** ^1^Department of Psychology, Utah State UniversityLogan, UT, USA; ^2^Department of Psychology, Washington State UniversityPullman, WA, USA; ^3^Department of Psychology, University Research Institute of Health Sciences (IUNICS), University of Balearic IslandsPalma, Spain

**Keywords:** multitrait-multimethod (MTMM) analysis, measurement invariance, measurement equivalence, mean and covariance structures, mean differences across raters, random vs. fixed methods, rater agreement

## Abstract

Models of confirmatory factor analysis (CFA) are frequently applied to examine the convergent validity of scores obtained from multiple raters or methods in so-called multitrait-multimethod (MTMM) investigations. We show that interesting incremental information about method effects can be gained from including mean structures and tests of MI across methods in MTMM models. We present a modeling framework for testing MI in the first step of a CFA-MTMM analysis. We also discuss the relevance of MI in the context of four more complex CFA-MTMM models with method factors. We focus on three recently developed multiple-indicator CFA-MTMM models for structurally different methods [the correlated traits-correlated (methods – 1), latent difference, and latent means models; Geiser et al., 2014a; Pohl and Steyer, 2010; Pohl et al., 2008] and one model for interchangeable methods (Eid et al., 2008). We demonstrate that some of these models require or imply MI by definition for a proper interpretation of trait or method factors, whereas others do not, and explain why MI may or may not be required in each model. We show that in the model for interchangeable methods, testing for MI is critical for determining whether methods can truly be seen as interchangeable. We illustrate the theoretical issues in an empirical application to an MTMM study of attention deficit and hyperactivity disorder (ADHD) with mother, father, and teacher ratings as methods.

Multitrait-multimethod (MTMM) analysis is frequently used to examine the convergent and discriminant validity of psychological measurements based on measurement designs in which multiple constructs or traits are assessed by multiple methods (Campbell and Fiske, 1959; Widaman, 1985; Millsap, 1995; Dumenci, 2000). In the classical MTMM design, multiple (typically at least three) methods are used to assess multiple (typically at least three) constructs or traits. The analysis of MTMM data has historically focused on the interpretation of the so-called *MTMM matrix*, which summarizes the correlations between variables in an MTMM design. The MTMM matrix approach was developed by Campbell and Fiske (1959) who also proposed heuristics for the interpretation of MTMM correlations in terms of convergent and discriminant validity. Over the years, confirmatory factor analysis (CFA) has become a popular tool for analyzing data obtained from MTMM designs, given the greater flexibility of the CFA framework compared to the original MTMM matrix approach (for a detailed discussion of the advantages of the CFA approach to MTMM analyses, see Eid et al., 2006).

Whereas Campbell and Fiske's original approach focused exclusively on correlation structures, CFA models allow analyzing not only correlation, but also covariance and mean structures (e.g., Little, 1997). Moreover, CFA models allow for the analysis of multiple (instead of just a single) indicators (e.g., items or scales) per trait-method unit (TMU). For example, self-, parent, and teacher-reports on three or more items or scales could be used to assess depression. Using multiple indicators per TMU has the advantage that researchers can study the factorial validity at the item level for each type of method, that method effects can be analyzed separately for different traits to examine the potential trait-specificity of method effects, and that measurement error influences (unreliability) can be more properly estimated (Marsh and Hocevar, 1988; Eid et al., 2003). Despite the fact that modern CFA methods allow for an analysis of covariance *and* mean structures in the same model, most applied MTMM studies so far have focused exclusively on modeling covariance structures. In addition, most MTMM studies still use single-indicator designs (i.e., just a single observed variable per TMU; e.g., Servera et al., 2010).

In the present article, we show that by moving from an exclusively covariance- or correlation-based MTMM approach to an approach that includes latent means, more fine-grained information about convergent validity and method effects can be gained in CFA-MTMM analyses. In this context, we highlight a specific advantage of multiple-indicator MTMM designs that has received little attention in the MTMM literature so far: the possibility to test for measurement invariance (MI) across multiple raters or methods when the different methods provided scores on comparable measurement instruments (e.g., equivalent questionnaires).

Analyzing mean structures in MTMM models and the investigation of mean method effects in MTMM models has been proposed in previous work (Eid, 2000; Pohl et al., 2008; Pohl and Steyer, 2010). The new aspect in the current paper is the investigation of MI across methods, which facilitates a proper interpretation of mean method effects. Examining MI is a novel aspect in MTMM research and considering MI itself as well as for the interpretation of mean method effects adds important information when evaluating MTMM data.

Although some MTMM studies have examined MI in the context of multiple-group comparisons (i.e., for comparing measurement structures across different populations; e.g., Cole and Maxwell, 1985; Marsh et al., 1992), the issue of MI across methods within the same population seems to have received little attention in the literature. For example, although Woehr et al. (2005) tested for configural, metric, and residual invariance across different raters, they did not examine intercept invariance or latent mean differences across raters.

In the present paper, we focus on MTMM designs that (1) use multiple raters as methods and (2) equivalent questionnaires across raters. Such designs are common in the applied MTMM literature. For example, Cole et al. (1997) used equivalent child and parent versions of questionnaires measuring depression and anxiety in children. Similarly, Grigorenko et al. (2010) used self-report, parent-report, and teacher-report versions of the same questionnaire to assess problem behaviors in children. Burns et al. (in press) assessed symptoms of hyperactivity, impulsivity, inattention, and academic impairment in 5th graders by mother, father, and teacher ratings, all of which filled out equivalent forms of a questionnaire.

We show that by studying MI across raters, additional information about method effects can be obtained that cannot be revealed through purely correlational MTMM analyses. By testing for MI, researchers can first of all examine whether the same factor structure holds across methods (configural invariance, see discussion below)—an assumption that is often implicitly made in MTMM studies, but rarely formally tested. In addition, researchers can examine whether different methods (e.g., different raters) use the questionnaire scales in a similar way (i.e., whether the scales have equal difficulty and discrimination across raters in the sense of item response theory). For example, when the same symptoms of attention deficit and hyperactivity disorder (ADHD) are rated by parents and teachers, different loadings or intercepts may be obtained, showing that the observed symptom scores differ in difficulty or discrimination between raters. This could, for example, indicate that teachers are more lenient than parents in their ratings or that certain symptoms are only weakly related to the latent variable for a specific type of rater. Therefore, the finding of measurement non-invariance across raters can reveal additional insights into more subtle forms of method effects.

In addition to the general relevance of MI testing across methods, researchers may be uncertain as to the relevance of MI in different CFA-MTMM models with method factors. With the present article, we also want to contribute to a better understanding of the issue of MI in the context of recently developed CFA-MTMM models. In line with modern MTMM approaches, we focus on models that use multiple indicators per TMU (Marsh and Hocevar, 1988; Eid et al., 2003, 2008; Geiser et al., 2012).

We first explain why the inclusion of means in addition to covariances and testing for MI can reveal useful incremental information in MTMM studies in general. We then present a modeling framework for testing MI in MTMM studies that use multiple indicators per TMU. Subsequently, we turn to four different models with method factors that have recently been proposed for the analysis of MTMM data. For each of the four models, we discuss which level of MI these models require for a proper interpretation of the model parameters.

## Analyzing mean structures in MTMM analyses: interesting incremental information

The reported outcome of most MTMM studies are statistical indices that provide information on the convergent validity (or consistency) of different methods or raters in terms of the rank order of the individuals that were assessed by the different methods. As a simple example, researchers often interpret a correlation between, say parent and teacher ratings of child behavior in terms of convergent validity, following Campbell and Fiske's (1959) guidelines. In terms of individual differences, such a correlation coefficient indicates to which extent different raters agree as to the rank order of children on the outcome variable (e.g., depression, externalizing problem behavior, ADHD). This information is clearly useful, as it informs us about how much variability is shared between raters or methods for the same construct.

Here, we argue that covariance-based information on multiple raters' agreement as to the relative standing of individuals on a construct (which is typically the focus of MTMM studies) is not the only useful information that can be gained from MTMM studies. This is because information about the overall level (mean) is usually also of interest. That is, we argue that researchers often want to know, for instance, whether parent ratings of problem behaviors result in the same or similar conclusions about the overall level of these behaviors in a population as do teacher ratings. Such questions can be addressed by analyzing mean structures in CFA-MTMM models in addition to covariance structures, which is a relatively novel aspect in MTMM research. Comparing means across raters requires a certain level of MI across raters. That is, for such comparisons to be meaningful, the measurement parameters that link the observed scores to the latent variables should be equal across raters to ensure comparable scales. This issue parallels the comparison of latent means across groups in multigroup CFA and structural equation modeling (SEM) as well as the examination of mean changes across time in longitudinal studies (e.g., Little, 1997).

## The meaning of MI for MTMM data

Formally, MI can be said to hold in an MTMM study if (1) a similar factor structure is found for different methods used to assess multiple traits or constructs using multiple indicators per TMU and/or (2) certain parameters of the measurement model (e.g., factor loadings, intercepts, or residual variances) that relates the observed scores to latent variables are equal across methods. Condition (1) requires only that (a) the same number of factors be found across methods and (b) the pattern of loadings (which variable loads onto which factor) be the same across methods. For Condition 1, the term *configural invariance* has been coined in the general MI literature (e.g., Meredith, 1993; Widaman and Reise, 1997; Millsap, 2011). Condition 2 is more restrictive and requires that not only the basic factor structure be equivalent across methods, but also specific parameters such as factor loadings, intercepts, or residual variances.

Even though it seems clear that establishing at least configural invariance (equal factor structure) across methods is a necessity for a meaningful comparison across methods, even configural invariance is typically not formally tested in MTMM studies (for exceptions, see Woehr et al., 2005; Burns et al., in press). Here, we argue that testing for MI is useful when different methods were scored on comparable scales (e.g., multiple raters taking the same questionnaire), because such analyses (1) provide additional insights into method effects and (2) allow researchers to test whether it is meaningful to compare latent means across raters. A meaningful comparison of latent means across methods requires that at least strong MI be established across methods (i.e., equal loadings and intercepts). Strong MI ensures that the origin and units of measurement are the same across raters.

## A modeling framework for testing MI in MTMM studies

**Figure 1:**
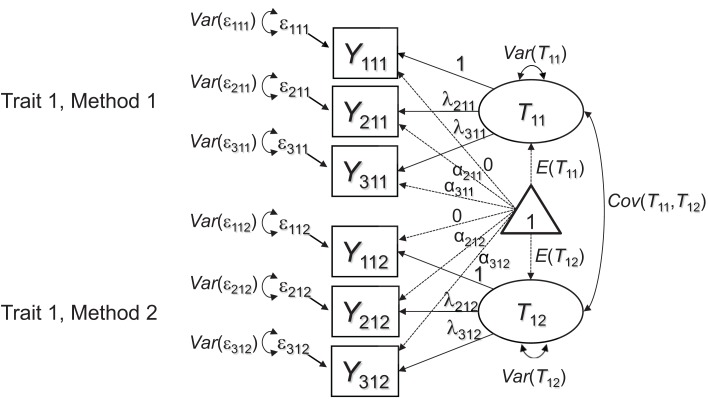
**CFA measurement model for multiple-indicator MTMM data**. Each latent factor *T_jk_* represents the error-free (true) scores of a specific TMU. The picture shows an example in which three indicators *Y_ijk_* (*i* = 1, 2, 3) are used to measure one construct or trait (*j* = 1) by two methods (*k* = 1, 2).

Marsh and Hocevar (1988) proposed a general target or baseline model for MTMM studies that use multiple indicators per TMU. In the present article, we show that this model as well as an extension of it can be used for testing MI across methods in MTMM studies. Marsh and Hocevar's model is depicted in Figure 1 as a path diagram. For simplicity, here we consider only a single construct (or trait; *j* = 1) that is measured by just two methods (*k* = 1, 2). Each TMU *jk* is represented by three indicators (*i* = 1, 2, 3). Focusing on this simple design is sufficient to explain the general MI issues, which can then easily be generalized to larger MTMM designs. (In our empirical application presented later on, we used a design with one trait and three methods).

Note that in our path diagrams, we represent both the covariance and mean structure, following the RAM conventions introduced by McArdle (1980). The model proposed by Marsh and Hocevar (1988) includes a separate common factor (or true score variable) for each TMU (e.g., one factor for mother ratings of hyperactivity and one factor for teacher ratings of the same construct). All TMU factors are allowed to correlate. Marsh and Hocevar's model has a number of advantages for MTMM analyses in general. First, the model allows testing the appropriateness of the latent factor structure for each TMU. For example, the assumption of unidimensionality may be violated for some or all methods, thus providing evidence against configural invariance across methods, which is fundamental to MTMM analysis. Second, the model allows examining Campbell and Fiske's (1959) MTMM correlations at the latent level. That is, rather than inspecting observed correlations that are attenuated by measurement error as in Campbell and Fiske's original approach, the model in Figure 1 provides the same correlations at the level of common true score variables. Therefore, the MTMM correlations are corrected for random measurement error. This has the advantage that the estimated correlations are less biased and easier to compare between constructs with different scale reliabilities^1^.

In the present article, we focus on the possibility to formally test for MI across methods within each construct or trait by estimating constrained versions of the model. In these constrained versions, parameters of the measurement model such as loadings, intercepts, or residual variances are constrained to be equal across methods to test whether and to which extent such MI assumptions are tenable. In order to test for MI, we can examine the following series of models in line with Widaman and Reise (1997)^2^:

1. A model of configural invariance postulates the same factor structure (number of factors and pattern of loadings) across methods, but does not impose any formal equality constraints on non-zero factor loadings, intercepts, or residual variances.
2. A model of weak invariance postulates the same factor structure plus equal factor loadings for corresponding indicators across methods.
3. A model of strong invariance postulates the same factor structure, equal factor loadings, and equal intercepts for corresponding indicators across methods.
4. A model of strict invariance postulates the same factor structure, equal factor loadings, equal intercepts, and equal measurement error (residual) variances for corresponding indicators across methods.

One drawback of the model presented in Figure 1 is that it will only fit MTMM data when the indicators are strictly homogeneous in the sense that within each method, all indicators have perfectly correlated true score variables that differ only in scaling (i.e., have potentially different intercepts and loadings). This assumption is frequently violated in practice, because different items or subscales often measure slightly different facets of a construct and therefore do not share exactly the same common true score variable in the sense of classical test theory models. For example, one item for measuring the construct depression may refer to *sadness*, whereas another item meant to measure the same construct may refer to *sleeping problems*. Therefore, although both items measure facets of depression, they may not share exactly the same true score variable. As another example, consider item wording effects due to positive and negative item wording (e.g., Vautier et al., 2003), which can also cause a common true score model to show misfit.

**Figure 2:**
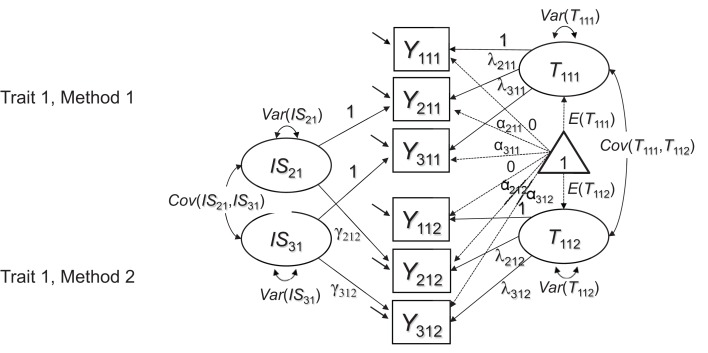
**Extended CFA measurement model for multiple-indicator MTMM data**. In contrast to Figure 1, the extended model contains *I – 1* indicator-specific factors *IS_ij_* to reflect shared indicator-specific effects across raters. The latent factors *T*_1*jk*_ are now specific to the reference indicator *Y*_1*jk*_ and therefore carry an additional index for the reference indicator.

If such inhomogeneities generalize across methods (e.g., if parent ratings of sadness are more strongly correlated with teacher ratings of sadness than with teacher ratings of sleeping problems), then the model in Figure 1 likely will not fit the data very well, because this model assumes a homogeneous correlation structure across methods for the same construct. We therefore present an extension of the model in Figure 1, in which this issue is addressed by including indicator-specific residual factors for all but a reference indicator (see Figure 2). An equivalent approach has been presented previously to account for indicator heterogeneity in longitudinal studies, in which the same issues occur when the same indicators are repeatedly measured across time in single-method designs (Eid et al., 1999).

The model in Figure 2 uses a reference-indicator approach in which *I* – 1 (of a total number of *I*) indicators are contrasted against a reference indicator (without loss of generality, the first indicator *i* = 1 in Figure 2 is chosen as reference indicator). This is done by introducing residual method (or indicator-specific) factors *IS_ij_* for all except the reference indicator. These indicator-specific factors have means of zero and are by definition uncorrelated with the true score that represents the reference indicator (see Appendix A in Supplementary Material for the formal definition of these factors). The *IS_ij_* factors reflect indicator-specific variance that is not shared with the reference indicator, but is shared across methods. Indicator-specific factors can be correlated with each other in principle, reflecting potential shared deviations of non-reference indicators from the reference indicator (e.g., the reference indicator measuring the sadness aspect of depression, whereas the remaining two indicators both refer to sleeping problems). Whether or not these correlations are meaningful and should be estimated depends on the specific application.

The model with indicator-specific factors can be used for MI testing in the same way as the model in Figure 1. If at least strong MI (i.e., equal reference factor loadings λ_*ijk*_ and intercepts α_*ijk*_) can be established in either the model in Figure 1 or the model in Figure 2, latent mean differences across methods can be meaningfully interpreted^3^. In the model with indicator-specific factors, it is also possible to test for invariant loadings γ_*ijk*_ of the *IS_ij_* factors (in addition to the reference factor loadings λ_*ijk*_).

Establishing invariant *IS_ij_* factor loadings is not necessary for a meaningful comparison of the reference factors *T*_1*jk*_ across methods (for more detailed explanations see Appendix A in Supplementary Material). In many applications, only the factors *T*_1*jk*_ and their invariance across methods will be of substantive interest. Nonetheless, comparisons of the γ_*ijk*_ loadings across methods can reveal interesting information about the extent to which indicator-specific effects are reflected in different methods. For example, some methods may not be as sensitive to subtle differences in item content as others. This can be reflected in non-invariant γ_*ijk*_ loadings across methods. In the following section, we examine the issue of MI in the context of more sophisticated CFA-MTMM models with method factors that researchers often use in a second step of an MTMM analyses. Subsequently, we present applications of all models to an actual data set.

## Different MTMM models

More sophisticated CFA-MTMM models are often employed because the simple CFA models in Figures 1, 2 do not directly express method effects in terms of latent variables (i.e., method factors), except for indicator-specific effects. In contrast, more complex CFA-MTMM models contain additional latent variables that directly reflect method effects in terms of latent methods factors. Such models allow explicitly contrasting different methods against a gold standard method (e.g., Eid, 2000; Pohl et al., 2008) or against a common trait (Pohl and Steyer, 2010). Furthermore, more complex models allow relating method effects to external variables, which is not possible in Marsh and Hocevar's (1988) simple CFA model discussed above.

In this article, we focus on four CFA-MTMM models that are relatively new: (1) Eid et al.'s (2003) multiple indicator CT-C(M – 1) model, (2) Pohl et al.'s (2008) latent difference model, (3) Pohl and Steyer's (2010) latent means model, and (4) Eid et al.'s (2008) CFA-MTMM model for interchangeable methods. Whereas the first three models were developed for use with structurally different methods (e.g., different fixed types of raters such as mothers, fathers, and teachers, which are not drawn from the same set of raters), Eid et al.'s (2008) CFA-MTMM model was developed for interchangeable (random) methods (e.g., randomly selected customers rating a product or service). Furthermore, whereas the first three models can all be defined as equivalent versions of Marsh and Hocevar's (1988) simple CFA model, the CFA-MTMM model for interchangeable raters in general implies a different covariance and mean structure.

**Figure 3:**
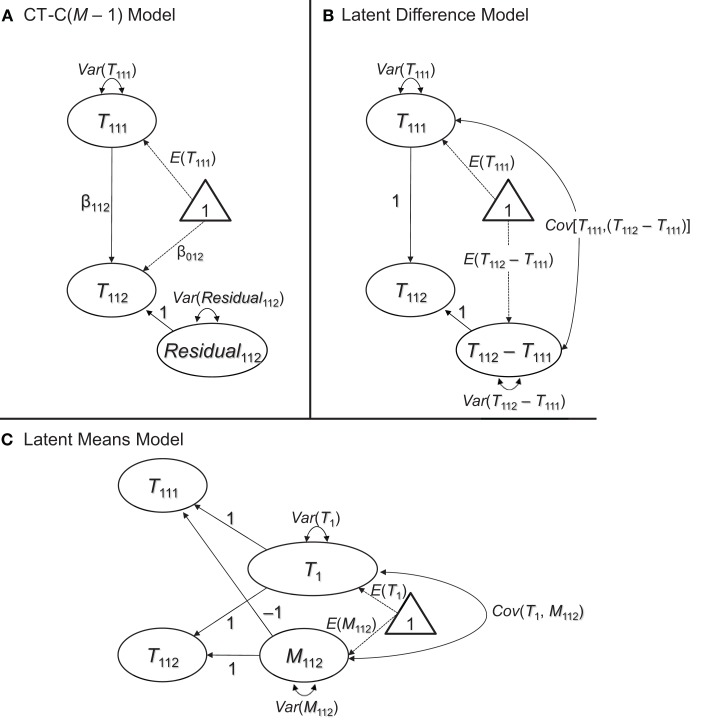
**Three different ways to examine method effects with structurally different methods. (A)** Latent regression [CT-C(*M* – 1)] approach. **(B)** Latent difference approach. **(C)** Latent means approach. All three approaches imply the same covariance and mean structure at the latent level, but differ in terms of which level of MI they require (see discussion in the text). The measurement part of the models is the same as in Figures 1, 2 and therefore not shown in this figure.

We focus on the above models, because all of them can be formulated based on the well-defined concepts of classical testy theory (CTT). This ensures that in all of the models, the trait and method factors have a clear meaning and interpretation (Geiser et al., 2014a). Given the fact that the CT-C(M – 1), latent difference, and latent means approaches each imply the exact same measurement model as the simple CFA model presented previously, we show only the structural parts of the models for simplicity and parsimony in Figure 3.

### The CT-C(M – 1) approach

#### Presentation of the model

Figure 3A shows the structural part of the CT-C(*M* – 1) model in the version first presented by Geiser et al. (2008) and discussed in detail in Geiser et al. (2012)^4^. In the CT-C(*M – 1)* model, one method serves as gold standard or reference method. This could either be a method that a researcher has most confidence in or that is most different from the remaining methods (for guidelines as to the choice of the reference method, see Geiser et al., 2008, 2012). For example, Geiser et al. (2014b) examined the convergent validity of giftedness assessments in children using the CT-C(*M* – 1) approach. They selected a maximum-performance test battery to serve as reference method, given that the test battery provided a more objective measure of abilities relative to more subjective ability ratings provided by the children themselves, their parents, and their teachers.

In Figure 3A, without loss of generality, the first method (*k* = 1) was chosen as reference. The second and any additional methods are regressed on the true score variable pertaining to the reference method using a latent regression analysis:

$$E\left(T_{1jk}|T_{1j1}\right)=\beta_{0jk}+\beta_{1jk}T_{1j1}$$

where β_0*jk*_ and β_1*jk*_ indicate regression coefficients and *k* ≠ 1. The residuals of these regressions

$${\text{Residual}}_{1jk}=T_{1jk}-E\left(T_{1jk}|\;T_{1j1}\right)$$

serve as method factors in the CT-C(*M* – 1) model. Note that the CT-C(*M* – 1) model has the same number of parameters in the structural model as the simple CFA model. For one construct and two methods, there are five structural parameters: the reference factor mean and variance, the regression coefficients β_0*jk*_ and β_1*jk*_, and the latent residual [method factor] variance. Note that for more than two traits or methods, admissible covariances among latent factors would be additional parameters to be estimated in the structural model.

Even though mathematically equivalent, the CT-C(*M* – 1) model represents a useful extension of the simple CFA model, because it allows us to express the information about method effects (defined relative to a reference method) in terms of latent method factors that are residuals with respect to the reference factors. Given that the method factors are defined as residuals relative to the reference factors, they are by definition uncorrelated with the reference factors and thus represent independent variance components (Eid et al., 2003). It also follows from their definition as residuals that the method factors have means of zero. Hence, it would not be meaningful to make statements about method factor means in the CT-C(*M* – 1) model.

The CT-C(*M* – 1) model allows us to (a) quantify what percentage of the observed or true score variance in different methods is shared vs. not shared with the reference method and (b) directly relate method effects to other variables (e.g., by correlating method factors with external variables). The proportion of observed variance that is shared with the reference method is expressed by the consistency coefficient:

$$Con\left(Y_{ijk}\right)\;=\lambda_{ijk}^{2}\beta_{1jk}^{2}Var\left(T_{1j1}\right)\;/\;Var\left(Y_{ijk}\right).$$

The consistency coefficient is often used as an indicator of convergent validity relative to the reference method or “gold standard.” The proportion of observed variance that is not shared with the reference method is expressed by the method-specificity coefficient:

$$MSpe\left(Y_{ijk}\right)\;=\lambda_{ijk}^{2}Var\left(Residual_{1jk}\right)\;/\;Var\left(Y_{ijk}\right).$$

The method-specificity coefficient is used to indicate which portion of the observed variance is unique to a specific method and not shared with the reference method. Correlations between method factors are allowed in the CT-C(*M* – 1) model. These correlations are partial correlations between non-reference methods from which variance shared with the reference method has been partialled out. Therefore, method factor correlations reflect a shared perspective (or “bias”) of non-reference methods relative to the reference method.

#### MI in the CT-C(M – 1) model

The CT-C(*M* – 1) model allows contrasting different methods against a reference method by means of a latent regression approach. For this purpose, strictly speaking, MI across methods beyond configural invariance is not required. That is, for the interpretation of the *standardized* regression coefficients as well as the coefficients of consistency and method-specificity, it does not matter whether different methods were measured on the same scale, because the coefficients of interest are standardized. This makes the CT-C(*M* – 1) model very flexible for examining the convergent validity of different methods. For example, Geiser et al. (2014b) examined the convergent validity of objective ability tests and subjective ability ratings. Objective and subjective assessments were made on completely different scales; nonetheless the CT-C(M – 1) model allowed examining the degree of convergent validity across these methods.

On the other hand, the interpretation of the *unstandardized* regression coefficients β_0*jk*_ and β_1*jk*_ can in some cases be difficult if the different methods used different scales. This issue parallels the potential difficulty of interpreting unstandardized regression coefficients in standard ordinary least squares regression analysis when predictor and criterion variables used different or arbitrary metrics. Furthermore, if a researcher wants to make comparisons of latent means across methods based on the latent mean of the reference factor and the unstandardized regression coefficients, strong MI is required in the same way as in Marsh and Hocevar's (1988) model.

### The latent difference approach

#### Presentation of the model

The latent difference approach is closely related to the CT-C(*M* – 1) approach in that different methods are contrasted against a reference method. However, in the latent difference approach, method effects are defined as simple deviations (differences) from a reference method true score variable rather than as regression residuals (Pohl et al., 2008). Latent difference factors (*T*_1*jk*_ − *T*_1*j*1_) are introduced that reflect method effects in terms of the difference between a true score of a non-reference method and the true score pertaining to the reference method (see Figure 3B):

$$T_{1jk}=\;1T_{1j1}+\;1\left(T_{1jk}-T_{1j1}\right).$$

The latent difference model again has the same number of parameters in the structural model as Marsh and Hocevar's (1988) simple CFA model (five parameters in the case of two methods: the reference factor mean and variance, the latent difference factor mean and variance, and the covariance between reference and latent difference factor). In contrast to the CT-C(*M* – 1) model, a correlation between reference and method factor is allowed in the latent difference model, because the method factor is not defined as a regression residual with respect to the reference factor. Moreover, in contrast to the CT-C(M – 1) approach, the mean of the method factor can be estimated as well and reflects the latent mean difference between two methods. A more detailed comparison of the latent difference and CT-C(*M* – 1) models can be found in Geiser et al. (2012).

#### MI in the latent difference model

In the latent difference model, convergent validity is assessed in terms of the latent difference between true score variables pertaining to different methods. Smaller differences indicate greater convergent validity relative to the reference method. MI plays a more important role in the latent difference model than in the CT-C(*M* – 1) model. Given that method effects are defined in terms of difference scores between the true score variables pertaining to different methods, strong MI is critical for a meaningful interpretation of the structural model parameters in the latent difference model. When strong MI does not hold, the interpretation of the latent difference scores can become difficult, because a violation of strong MI indicates that the true score variables pertaining to different methods may not be measured with comparable origin or units of measurement. In this case, persons' individual difference scores as well as the mean and variance of the latent difference factor would be difficult to interpret.

### The latent means approach

#### Presentation of the model

In the latent means model, method effects are defined as deviations from an average across true score variables (Pohl and Steyer, 2010). In the first step, a common trait factor *T_j_* is defined by averaging across the true score variables that reflect different TMUs. In our example with just one trait and just two methods, we obtain:

$$T_{j}:=\;\left(T_{1j1}+T_{1j2}\right)\;/\;2,$$

where the “:=” sign indicates a definition. The method factors are defined as deviations from the common trait:

$$\begin{array}{l} M_{1j1}:=T_{1j1}-T_{j},\\ M_{1j2}:=T_{1j2}-T_{j}. \end{array}$$

Given their definition as deviations from the same average, the method factors sum up to zero (i.e., *M*_1*j*1_ + *M*_1*j*2_ = 0). Therefore, in the case of two methods, we obtain the following deterministic relationship between the two method factors:

$$M_{1j1}=-M_{1j2}.$$

It is thus sufficient to include only *M* – 1 method factors as in the CT-C(*M* – 1) and latent difference models (i.e., the last method factor is fully determined by the implicit sum-to-zero constraint and therefore redundant). Here, without loss of generality, we dropped the first method factor so that we obtain the following structural model shown in Figure 3C:

$$\begin{array}{l} T_{1j1}=T_{j}-M_{1j2},\\ T_{1j2}=T_{j}+M_{1j2}. \end{array}$$

All trait and all method factors in the latent means model can be correlated. As in the CT-C(*M* – 1) and latent difference models, for two TMUs, we obtain a structural model with five free parameters (the common trait factor mean and variance, the method factor mean and variance, and the covariance between the common trait and the method factor).

#### MI in the latent means model

The latent means model defines a common trait *T_j_* as the average of true score variables *T*_1*jk*_ that pertain to the same construct *j*. Such an average is typically only meaningful when the true score variables are measured on the same scale. A similar argument applies to the interpretation of the method factors in the model: A deviation of a particular method from the grand average is only meaningful if all methods used the same scale. Moreover, as in the latent difference model, establishing at least strong MI is crucial for the interpretation of the model parameters in the latent means model. One difference between the latent difference and latent means models in this regard is that the latent difference model allows for partial MI, whereas the latent means model does not. That is, as long as at least one non-reference method shows MI relative to the reference method, the latent difference between the two can be meaningfully interpreted. In the latent means model, however, the common trait will typically only have a clear interpretation if *all* methods show at least strong MI.

### The CFA-MTMM model for interchangeable methods

#### Presentation of the model

Eid et al. (2008) showed that measurement designs with interchangeable methods imply different measurement models for modeling trait and method effects than do designs with structurally different methods. This is because the underlying random experiment differs for designs with structurally different vs. interchangeable methods. Eid et al. (2008) presented a multilevel CFA approach for modeling interchangeable methods each of which used the same items to rate each trait. Nussbeck et al. (2009) showed that the same model can also be estimated within the single-level CFA framework. Here, we consider Nussbeck et al.'s single-level CFA approach (rather than the multilevel version) for two reasons: (1) The single-level version of the model is easier to compare to the previously described models for structurally different methods, and (2) the single-level CFA approach is more flexible in terms of explicitly testing assumptions of MI than is the multilevel approach (this parallels the issue of testing MI in longitudinal latent state-trait models in the single- vs. multilevel CFA framework as described in detail in Geiser et al., 2013).

**Figure 4:**
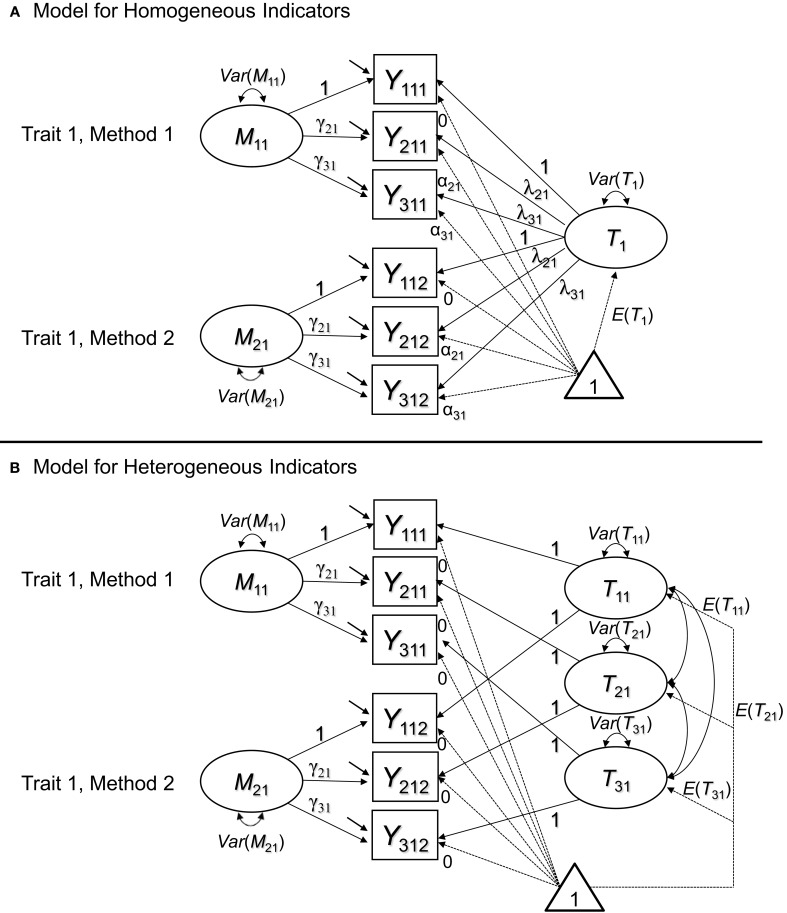
**CFA-MTMM models for interchangeable methods. (A)** Version with a general trait factor for homogeneous indicators. **(B)** Version with indicator-specific traits for heterogeneous indicators. In the pictures, we show the recommended specification, in which loadings and intercepts are set equal across methods for identical indicators. In model version B, all trait factor loadings are fixed to 1 and all intercepts are fixed to zero.

When methods are interchangeable, they are considered randomly drawn from a set of equivalent methods (Eid et al., 2008). Eid et al. (2008) as well as Nussbeck et al. (2009) showed that this structure implies a CFA model with *M* uncorrelated method factors (i.e., a separate method factor for each interchangeable method; see Figure 4). As can be seen in Figure 4, for one trait and two methods, we obtain measurement models that have the same structure as longitudinal latent state-trait models (e.g., Geiser et al., 2013) for multiple indicators and that are similar to so-called bifactor models (e.g., Reise, 2012).

Figure 4A is a version of the model for homogeneous indicators all of which measure exactly the same common trait factor *T_j_*. Figure 4B shows a model version with indicator-specific trait factors *T_ij_*. Indicator specific traits are useful to capture inhomogeneities among indicators in a similar way as was done with indicator-specific factors in the previously discussed models for structurally different methods. The method factors *M_jk_* are defined as residuals with respect to the trait factor(s). As a consequence, the trait factors are by definition uncorrelated with all *M_jk_* pertaining to the same construct *j*, and all *M_jk_* factors have means of zero by definition. Similar to the CT-C(*M* – 1) model, method effects are defined as regression residuals. In contrast to the CT-C(*M* – 1) model, however, the trait factors are common to all methods (rather than specific to a reference method), and the method factors are uncorrelated across methods. This makes sense, because of the interchangeable nature of the ratings. In contrast to structurally different methods, with interchangeable methods, there is no one method that is particularly outstanding or special (or seen as a gold standard). Therefore, it makes sense to include general trait factors and uncorrelated method factors for each method.

Note that the common factors and the method factors in the interchangeable model have a different meaning than the trait and method factors in the latent means model. The common trait in the latent means model is defined as an average of true scores, and the method factors are defined as differences from this average. In contrast, the common factor in the interchangeable model is not an average and the method factors are not differences from an average, but residuals with respect to a common factor.

#### MI in the CFA model for interchangeable methods

The interchangeable model is an interesting case with regard to the issue of MI, because it is less obvious whether or not MI across methods is required or should be established in the model. For the interpretation of the traits and method factors in the model, MI does not appear to be necessary, because the traits are not defined as an average of true scores, and the method factors are not defined as difference scores relative to a reference true score or an average of true scores as in the latent difference and latent means models. Nonetheless, testing for invariant loadings and intercepts across methods is critical in this model as well, albeit for different, somewhat more subtle reasons.

If different supposedly interchangeable methods result in different loadings or intercepts for the same indicator, this can question the assumption that these methods are truly interchangeable in the sense that they represent “random samples” drawn from a set of uniform methods. For example, a researcher may ask a person to come to a laboratory and bring two randomly selected friends to provide ratings of the person with respect to psychological variables (e.g., personality variables such as agreeableness etc.). The target person may not be sure whom to bring and may select his or her best friend as well as a more distant acquaintance. In this case, the “best friend” may have access to different information about the target person than the acquaintance. Hence, the two ratings may be considered structurally different rather than interchangeable. Non-interchangeable ratings may result in, for example, different latent means. This may result in a misfit of a model with equal loadings or intercepts, providing evidence against the assumed interchangeable nature of the two raters.

Strictly speaking, a researcher dealing with truly interchangeable methods (in the sense that the ratings represent random draws from a population of equivalent methods) would not only expect to find equal trait and method factor loadings as well as equal intercepts across methods, but also equal error and equal method factor variances for each type of rater.

Obviously, the issue of MI in the CFA-MTMM model for interchangeable methods can often be resolved by making sure the methods are truly selected at random from a set of uniform methods. In this case, by definition, the measurement parameters are equivalent in the population (although they may differ in a sample due to sampling fluctuations). However, in practice, a random selection of raters or other methods may not always be feasible. Tests of MI can then provide a way to scrutinize whether the assumption of interchangeable methods is warranted or whether the chosen methods should better be treated as structurally different. In the latter case, the use of one of the three previously discussed models for structurally different methods would be preferable. Below we present an application of all five models to an MTMM study on ADHD symptoms.

## Empirical illustration

### Sample and measures

The participants were mothers, fathers, and teachers of 1045 first grade children from 22 randomly selected elementary schools on the island of Majorca in the Balearic Islands and eight schools from Madrid (Spain). Assessments one and two occurred in the spring with assessment three occurring 12-months later. For the present illustration, we used data from the third assessment for which *N* = 709 (HI; *j* = 1) and *N* = 710 (IN; *j* = 2) cases with mother ratings (*k* = 1), father ratings (*k* = 2), and teacher ratings (*k* = 3) were available. The average age of the children was approximately 8 years with approximately 90% of the children being Caucasian and 10% North African.

Mothers, fathers, and teachers completed Child and Adolescent Disruptive Behavior Inventory (CADBI, Burns and Lee, 2010a,b). This study used the nine symptoms on the attention-deficit/hyperactivity disorder-inattention (ADHD-IN) and the nine symptoms on the ADHD-hyperactivity/impulsivity (HI) subscales. The ADHD symptoms were rated on a 6-point scale [i.e., *nearly occurs none of the time (e.g., 2 or fewer times per month)*, *seldom occurs (e.g., once per week)*, *sometimes occurs (e.g., a few times per week)*, *often occurs (e.g., once per day)*, *very often occurs (e.g., several times per day)*, and *nearly occurs all the time (e.g., many times per day)*].

For the purpose of this demonstration, item parcels were used as indicators rather than individual symptoms, given that earlier research provided justification for the use of parcels (Burns et al., in press).

### Modeling strategy

In Step 1 of our analyses, we attempted to establish a well-fitting baseline model for conducting subsequent MI analyses. For this purpose, we fit both Marsh and Hocevar's (1988) simple CFA model (Figure 1) and the extended model with indicator-specific factors (Figure 2) to the data and compared their fit to test whether homogeneity of the indicators (parcels) as well as configural invariance (equal factor structure) across raters could be assumed in the present application. None of the initial models included any formal equality constraints on measurement parameters. If the model in Figure 1 for homogeneous indicators had fit the data well, it would have been the preferable model for further invariance tests relative to the more complex model in Figure 2, because the latter model is less parsimonious. In case of a substantially better fit of the more complex model in Figure 2, the more complex model is preferred, indicating a certain degree of indicator heterogeneity.

In Step 2, we proceeded with tests of MI across raters, using the best-fitting model from Step 1. The analyses in Step 2 began with a model of weak factorial invariance (only equal loadings across raters), then tested a strong invariance model (equal loadings and equal intercepts across raters), and finally a model of strict invariance (equal loadings, equal intercepts, and equal residual variances across raters). Given that all subsequent models were nested within previous models, we performed chi-square difference tests to compare the fit of the models directly. In cases in which one of the subsequent models showed a significantly worse fit than the preceding model, we further investigated issues of partial MI. That is, we tested in these cases whether there was invariance across some of the raters (e.g., mother and father, but not teacher ratings). Given that mother and fathers rated the children in the same context (at home), our hypothesis was that mother and father ratings may satisfy a stricter level of MI than parent and teacher ratings. In the final step, we tested for latent mean differences across raters if at least strong MI could be established for at least one pair of raters (e.g., mother and father ratings). In Step 3, we fit more complex CFA-MTMM models with method factors if this was warranted given the level of MI achieved in Steps 1 and 2.

All models were fit in Mplus 7 using maximum likelihood estimation. Examples of the Mplus specification for all models can be found in Appendix B in Supplementary Material. Global model fit was evaluated using the chi-square test, root mean square error of approximation (*RMSEA*), comparative fit index (*CFI*), Tucker-Lewis index (*TLI*), and standardized root mean square residual (*SRMR*). For a review and detailed discussion of these fit indices, see Schermelleh-Engel et al. (2003). Relative model fit was assessed via the chi-square difference test for nested models and Akaike's information criterion (*AIC*).

### Results of the MI analyses

Table 1 shows global and relative model fit statistics for both the HI and IN constructs in the analyses of the Figure 1, 2 models. It can be seen that for both HI and IN, based on global model fit and the *AIC*, Model 1 (the configural invariance model without indicator-specific factors) clearly had to be rejected in favor of Model 2 (configural invariance with indicator-specific factors) as a baseline model. The configural invariance model with indicator-specific factors fit the data very well overall, showing a non-significant chi-square value for both HI and IN as well as excellent results based on other fit indices. An inspection of the model parameters revealed that for both HI and IN, the indicator-specific factors had significant (albeit relatively small) loadings (see Table 2), showing that the parcels were essentially, but not perfectly homogeneous. We therefore used the model with indicator-specific factors as the baseline model for subsequent MI tests involving equality constraints on loadings, intercepts, residual variances, and latent means for both HI and IN.

**Table 1:** **Goodness of fit statistics for different models fit to the HI and IN multirater data set**.

**Model**	**χ^2^**	***df***	***p***	***RMSEA***	***CFI***	***TLI***	***SRMR***	**χ^2^Δ**	***df*Δ**	***p*(χ^2^Δ)**	***AIC***
**HYPERACTIVITY/IMPULSIVITY**											
Figure 1 configural invariance	269.91	24	<0.001	0.12	0.963	0.944	0.02				9773
Figure 2 configural invariance	17.70	17	0.41	0.01	1.000	1.000	0.01	—^a^	—^a^	—^a^	9535
Figure 2 weak invariance	21.40	21	0.44	0.01	1.000	1.000	0.02	3.7	4	0.45	9531
Figure 2 strong invariance	29.85	25	0.23	0.02	0.999	0.999	0.02	8.45	4	0.08	9531
Figure 2 strict invariance	52.60	31	0.009	0.03	0.997	0.996	0.02	22.75	6	<0.001	9542
Figure 2 strong invariance with equal means	104.27	27	<0.001	0.06	0.988	0.984	0.08	74.42^b^	2^b^	<0.001^b^	9602
Figure 2 strong invariance with equal means only for mother and father reports	**30.52**	**26**	**0.25**	**0.02**	**0.999**	**0.999**	**0.02**	**0.67^b^**	**1^b^**	**0.41^b^**	**9530**
**INATTENTION**											
Figure 1 configural invariance	196.64	24	<0.001	0.10	0.975	0.962	0.02				9386
Figure 2 configural invariance	16.09	17	0.52	0.00	1.000	1.000	0.01	—^a^	—^a^	—^a^	9219
Figure 2 weak invariance (all raters)	28.18	21	0.14	0.02	0.999	0.998	0.02	12.09	4	0.02	9223
Figure 2 weak invariance (mothers and fathers only)	17.66	19	0.55	0.00	1.000	1.000	0.01	1.57^c^	2^c^	0.46^c^	9217
Figure 2 strong invariance (all raters)	193.63	25	<0.001	0.10	0.976	0.965	0.04	165.45	4	<0.001	9381
Figure 2 strong invariance (mothers and fathers only)	29.20	23	0.17	0.02	0.999	0.999	0.02	11.54^d^	4^d^	0.02^d^	9220
Figure 2 strict invariance (all raters)	230.73	31	<0.001	0.10	0.971	0.966	0.04				9406
Figure 2 strict invariance (mothers and fathers only)	**30.39**	**26**	**0.25**	**0.02**	**0.999**	**0.999**	**0.02**	**1.19^e^**	**3^e^**	**0.76^e^**	**9215**
Figure 2 strict invariance across mothers and fathers only with equal means only for mother and father reports	34.81	27	0.14	0.02	0.999	0.998	0.02	4.42	1	0.04	9218

*Note: RMSEA, root mean square error of approximation; CFI, comparative fit index; TLI, Tucker-Lewis index; SRMR, standardized root mean square residual; AIC, Akaike's information criterion. All chi-square difference tests refer to the previous model in the preceding row unless otherwise indicated. Bold-face indicates best-fitting models for which detailed results are presented*.

a *No chi-square difference test reported, because model nesting involves boundary constraints in this case*.

b *Relative to the strong invariance model with unequal means*.

c *Relative to the configural invariance model*.

d *Relative to the model of weak invariance for mother and father reports only*.

e *Relative to the model of strong invariance for mother and father reports only*.

**Table 2:** **Parameter estimates of the measurement models fit to the HI and IN multirater data set**.

**Parameter label**	**Hyperactivity/impulsivity (*j* = 1)**			**Inattention (*j* = 2)**		
	**Estimate**	***SE***	**Standardized estimate**	**Estimate**	***SE***	**Standardized estimate**
**TRAIT FACTOR LOADINGS**						
λ_1*j*_	1.00^a^	—	0.96^b^; 0.95^b^; 0.97^b^	1.00^a^	—	0.96^b^; 0.96^b^; 0.98^b^
λ_2*j*_	0.93	0.01	0.90^b^; 0.90^b^; 0.94^b^	0.13	0.01	0.91^b^; 0.92^b^; 0.96^b^
λ_3*j*_	0.92	0.01	0.90^b^; 0.90^b^; 0.94^b^	0.95	0.01	0.88^b^; 0.89^b^; 0.93^b^
**INDICATOR-SPECIFIC FACTOR LOADINGS**						
γ_2*j*1_	1.00^a^	—	0.37	1.00^a^	—	0.33
γ_2*j*2_	0.88	0.21	0.34	0.96	0.08	0.31
γ_2*j*3_	0.17	0.06	0.07	0.31	0.07	0.09
γ_3*j*1_	1.00^a^	—	0.24	1.00^a^	—	0.35
γ_3*j*2_	1.57	0.56	0.40	0.94	0.08	0.32
γ_3*j*3_	0.13	0.08	0.03	0.06	0.08	0.02
**INTERCEPTS**						
α_1*j*1_	0.00^a^	—		0.00^a^	—	
α_2*j*1_	−0.09	0.02		0.06	0.02	
α_3*j*1_	0.08	0.02		0.16	0.02	
α_1*j*2_	0.00^a^	—		0.00^a^	—	
α_2*j*2_	−0.09	0.02		0.06	0.02	
α_3*j*2_	0.08	0.02		0.16	0.02	
α_1*j*3_	0.00^a^	—		0.00^a^	—	
α_2*j*3_	−0.09	0.02		0.01	0.02	
α_3*j*3_	0.08	0.02		−0.15	0.02	
**ERROR VARIANCES**						
*Var*(ε_1*j*1_)	0.09	0.01	0.08^c^	0.07	0.01	0.09^c^
*Var*(ε_2*j*1_)	0.07	0.04	0.06^c^	0.08	0.01	0.07^c^
*Var*(ε_3*j*1_)	0.15	0.02	0.13^c^	0.09	0.01	0.11^c^
*Var*(ε_1*j*2_)	0.10	0.01	0.09^c^	0.07	0.01	0.08^c^
*Var*(ε_2*j*2_)	0.09	0.03	0.08^c^	0.08	0.01	0.07^c^
*Var*(ε_3*j*2_)	0.02	0.06	0.02^c^	0.09	0.01	0.10^c^
*Var*(ε_1*j*3_)	0.07	0.01	0.06^c^	0.06	0.01	0.05^c^
*Var*(ε_2*j*3_)	0.11	0.01	0.11^c^	0.10	0.01	0.06^c^
*Var*(ε_3*j*3_)	0.13	0.01	0.12^c^	0.15	0.01	0.13^c^

*Note: For hyperactivity/impulsivity, a model of strong invariance for all raters and equal means across mother and father ratings was chosen. For inattention, a model of strict invariance for mother and father ratings was chosen. λ_ijk_, trait factor loading (i, indicator; j, trait; k, method/rater); γ_ijk_, indicator-specific factor loading; α_ijk_, intercept; Var(ε_ijk_), error variance. The methods used here are mother report (k = 1), father report (k = 2), and teacher report (k = 3)*.

a *Parameter fixed for identification*.

b *Standardized loadings differed between raters for the same variable, because error variances and latent factor variances were allowed to differ in the final models. The standardized loadings are therefore given separately for each rater type in the following order: (1) mothers, (2) fathers, (3) teachers*.

c *Standardized residual variances indicate 1 – R^2^ and can be interpreted as coefficients of unreliability [(1 – Rel(Y_ijk_)] for each variable*.

*Dashes indicate fixed parameters for which no standard errors are computed*.

For HI, the assumptions of weak and strong MI across raters did not lead to a significant decline in model fit as indicated by chi-square difference tests^5^. The strong MI model also showed a very good global fit (non-significant chi-square). In contrast, the model of strict invariance was rejected by the chi-square difference test and also showed an increased *AIC* value relative to the strong invariance model. We concluded that error variances differed significantly across raters, whereas loadings and intercepts did not. Given that strong MI is sufficient for demonstrating scale equivalence and for meaningful comparisons of latent means, we proceeded with the strong-MI model and tested for latent mean differences across all three rater types for the HI construct.

The strong-MI model with equal means across all three rater types was clearly rejected for HI, showing that there were true mean differences across some of the raters (indicating a lack of convergent validity with respect to the true means or true mean differences between the home vs. school contexts). We additionally tested whether the means for mother and father ratings were significantly different from one another, or whether the parent means only differed from the teacher means. A model with latent means constrained equal across mother and father (but not teacher) ratings was not rejected by the chi-square difference test relative to the strong MI model with unconstrained latent means. Therefore, we concluded that mothers and fathers did show convergent validity of mean levels for the HI construct, whereas the latent mean for teacher ratings was significantly smaller than for mothers and fathers. This indicated a lack of convergent validity with regard to the HI mean level across parent and teacher ratings or true differences in the mean HI levels between contexts (home vs. school; more details are provided later on when we discuss the parameter estimates of the final models).

Our analyses of the IN construct yielded different findings with regard to MI. In the IN case, already the weak invariance model showed a statistically significant (albeit relatively modest) increase in the chi-square relative to the configural invariance model, indicating at least partly non-invariant loadings across some of the raters. We tested whether the loadings were equivalent at least across mother and father ratings, as mothers and fathers were rating the same context (home). This hypothesis was not rejected by the chi-square difference test.

The strong-MI model for all raters showed a marked and highly significant increase in the chi-square relative to the full weak invariance model. We again tested whether strong invariance could at least be assumed across mothers and fathers. This hypothesis was also rejected according to the chi-square difference test, although the resulting chi-square difference was relatively modest and the global chi-square for this model was still non-significant. The strong-MI model for mothers and fathers also showed a very good global model fit, as indicated by a non-significant overall chi-square goodness of fit test. We therefore decided to proceed with the “partial strong-MI” model and to also test for strict MI across mother and father ratings only (leaving the intercept and residual variance parameters for teachers unconstrained). The strict-MI model for mothers and fathers showed a good overall model fit in terms of the chi-square and did not fit significantly worse than the partial strong-MI model. We therefore used the partial strict-MI model to test for mean differences across mothers and fathers. The resulting model with equal means across mothers and fathers showed a significant, albeit rather small chi-square difference value, indicating that there was a small difference in the latent means between mother and father ratings of IN.

A key finding of the IN analyses was the clear non-invariance of teacher intercepts relative to mother and father intercepts for this construct. The parameter estimates for the final models (strong MI across all three raters and equal latent means across mother and father reports for HI; strict MI across mother and father ratings only and unconstrained latent means across all raters) are presented in Tables 2, 3. Table 2 contains the parameter estimates related to the measurement models (i.e., the loadings, intercepts, and error variances). Table 3 shows the structural (latent variable model) parameter estimates (i.e., the latent covariances, correlations, variances, and means).

**Table 3:** **Estimated latent covariances, correlations, means, and variances in the final models**.

	**1**.	**2**.	**3**.	**4**.	**5**.
**HYPERACTIVITY/IMPULSIVITY**					
1. *T*_111_	—	0.85 (0.06)	0.45 (0.05)	—^a^	—^a^
2. *T*_112_	0.81 (0.02)	—	0.43 (0.05)	—^a^	—^a^
3. *T*_113_	0.42 (0.04)	0.42 (0.04)	—	—	—^a^
4. *IS*_21_	—^a^	—^a^	—^a^	—	0.03 (0.01)
5. *IS*_31_	—^a^	—^a^	—^a^	0.29 (0.07)	—
Means	1.10^b^ (0.04)	1.10^b^ (0.04)	0.71 (0.04)	—^a^	—^a^
Variances	1.11 (0.07)	0.99 (0.06)	1.05 (0.06)	0.16 (0.04)	0.07 (0.03)
**INATTENTION**					
6. *T*_121_	—	0.60 (0.04)	0.41 (0.04)	—^a^	—^a^
7. *T*_122_	0.78 (0.02)	—	0.44 (0.05)	—^a^	—^a^
8. *T*_123_	0.45 (0.04)	0.44 (0.04)	—	—^a^	—^a^
9. *IS*_22_	—^a^	—^a^	—^a^	—	0.05 (0.01)
10. *IS*_32_	—^a^	—^a^	—^a^	0.44 (0.07)	—
Means	0.97 (0.04)	1.03 (0.04)	0.88 (0.04)	—^a^	—^a^
Variances	0.74 (0.05)	0.80 (0.05)	1.17 (0.07)	0.12 (0.02)	0.10 (0.02)

*Note: Covariances are shown above the diagonal, correlations below the diagonal*.

a *Covariances, correlations, or means that are set to zero by definition of the model. Standard errors are given in parentheses*.

b *Latent means for hyperactivity/inattention were set equal across mother and father reports*.

From Table 2, it can be seen that for IN, the intercepts for teacher ratings were markedly lower than the parent intercepts, indicating that teachers generally found it more “difficult” to endorse the symptoms on each of the IN indicators than did parents. One explanation could be that teachers in general are perhaps more used to seeing a broad spectrum of symptoms of IN and distraction in class than are parents at home. Therefore, the teachers in our sample may have used a different frame of reference (and a higher “threshold”) when making their ratings of IN compared to parents. As a consequence, a more serious level of observed IN symptoms was required for teachers to produce the same score on the latent variable of IN as would be obtained from parent ratings. Interestingly, this difference was only found for IN, but not HI. This shows us that MI analyses in the context of MTMM data can reveal quite interesting information about differences between methods and how they may or may not use rating scale in a different way that may lead to scores that are not directly comparable. This information goes beyond what is typically assessed in MTMM studies and what can be obtained from an MTMM matrix alone.

From Table 3, we can see that there was substantial convergent validity in terms of the rank order of children for both HI and IN. Latent correlations ranged between 0.78 and 0.81 for mother and father ratings and between 0.42 and 0.45 between parents and teachers. Latent means for mother and father ratings of HI were set equal, given that this constraint was supported by the goodness-of-fit tests. In contrast, teacher ratings of HI resulted in a significantly smaller latent mean than parent ratings (0.71 vs. 1.10). The standardized mean difference was about 0.35, which can be seen as a small effect. It could be that teachers again use a different frame of reference for problems of HI, as they may be used to seeing a much larger array of problem behaviors at school than what most parents experience at home. Another explanation could be that the possibly more structured school context relative to less structure at home reduces the occurrence of HI symptoms in school relative to home for children within the normal range on HI.

For IN, the latent mean based on father reports was slightly larger than the mother-report mean (1.03 vs. 0.97, which represented a standardized mean difference below 0.10 and hence a very small effect). Our MI analyses for IN had indicated that the teacher mean could not be directly compared to the parent means, given the finding of intercept non-invariance for teacher as compared to parent ratings.

#### CT-C(M – 1) model

Our analyses with the CT-C(*M* – 1) model allowed us to estimate the consistency and method-specificity coefficients relative to a reference method. For the present example, we chose to select mother reports (*k* = 1) as reference method and contrast father reports (*k* = 2) and teacher reports (*k* = 3) against this reference. This also made it possible to examine correlations between the father and teacher method factors, *Corr*(*M*_*j*2_, *M*_*j*3_). These correlations reflect whether fathers and teachers shared a common perspective that theses rater types did not share with mother reports.

Our results revealed high consistency and relatively low method-specificity coefficients for father reports for both HI and IN [range of consistency coefficients: 0.53 ≤ *Con*(*Y*_*i*12_) ≤ 0.60 for HI; 0.48 ≤ *Con*(*Y*_*i*22_) ≤ 0.55 for IN; range of method-specificity coefficients: 0.27 ≤ *MSpe*(*Y*_*i*12_) ≤ 0.31 for HI; 0.32 ≤ *MSpe*(*Y*_*i*22_) ≤ 0.36 for IN], indicating that there was high convergent validity between mother and father reports. This finding reflected the high correlations found between the mother and father trait factors in the baseline CFA model that we reported above. Consistency coefficients were lower (and method-specificity coefficients higher) for teacher ratings [range of consistency coefficients: 0.15 ≤ *Con*(*Y*_*i*13_) ≤ 0.16 for HI; 0.17 ≤ *Con*(*Y*_*i*23_) ≤ 0.19 for IN; range of method-specificity coefficients: 0.73 ≤ *MSpe*(*Y*_*i*13_) ≤ 0.77 for HI; 0.70 ≤ *MSpe*(*Y*_*i*23_) ≤ 0.76 for IN], showing that mother and teacher ratings shared less variance with each other than did mother and father ratings. The latent correlations between the father and teacher method factors were estimated to be φ = 0.15 for HI and φ = 0.19 for IN (both *p*-values were < 0.001). These correlations can be interpreted as partial correlations between father and teacher ratings after partialling out the common variance that both rater types shared with mother reports. In this case, the method factor correlations were rather small. This indicated that there was not much of a shared perspective between fathers and teachers above and beyond what fathers and teachers both shared with mothers.

#### Latent difference model

In the present example, a latent difference approach could be used for HI for all three rater types (mothers, fathers, and teachers), given that strong MI had been established across all three rater types for this construct. Given intercept non-invariance of teacher ratings as compared to parent ratings for IN, a latent difference approach would have been easily interpretable only for mother vs. father ratings for IN (excluding teacher ratings). We therefore only present the results for HI here as an example, for which we could meaningfully include all three rater types.

The latent difference model for HI yielded a latent difference factor mean of *E*(*T*_112_ – *T*_111_) = −0.02 [*Var*(*T*_112_ – *T*_111_) = 0.40] for father vs. mother ratings. This latent difference factor mean was not significantly different from zero (*p* = 0.41), showing that mother and father ratings of HI did not differ significantly in their latent means. The latent difference factor mean for teachers vs. mothers was estimated to be *E*(*T*_113_ – *T*_111_) = −0.40 [*Var*(*T*_113_ – *T*_111_) = 1.26], which was statistically significantly different from zero (*p* < 0.001). This again showed that mother and teacher ratings resulted in significantly different estimates of the overall level of HI in our data example. The correlations of father and teacher latent difference factors with the mother reference factor were φ = −0.40 and φ = −0.56, respectively. The latent difference factors for father and teacher ratings were correlated φ = 0.33.

#### Latent means model

The latent means model defines a common trait as the average across method-specific true score variables. Therefore, a latent means approach is only interpretable if *all* methods show at least strong MI. Otherwise, the “grand mean” of methods will be difficult to interpret. Therefore, we decided not to estimate the latent means model for IN because of intercept non-invariance for teacher ratings. The model was meaningful for HI, however, because all three rater types were shown to have invariant loadings and intercepts for this construct. The latent means model for HI yielded a common latent mean estimate of *E*(*T*_1_) = 0.97 [*Var*(*T*_1_) = 0.73]. The method factors in the latent means model represent deviations from the common latent mean factor. Their means indicate to which extent methods (on average) deviate from the grand mean across methods. The means of the method factors were estimated to be *E*(*M*_12_) = 0.12 [*Var*(*M*_12_) = 0.21] for father reports and *E*(*M*_13_) = −0.26 [*Var*(*M*_13_) = 0.50] for teacher reports. This reflected the fact that the latent mean of teacher ratings was substantially lower than the latent means for mother and father ratings of HI. The common trait was correlated φ = 0.06 with *M*_12_ and φ = −0.15 with *M*_13_. The correlation between *M*_12_ and *M*_13_ was estimated to be φ = −0.73.

#### CFA-MTMM model for interchangeable methods

We fit both the general and the indicator-specific trait versions of Eid et al.'s (2008) CFA-MTMM model for interchangeable methods to our data example to test whether the ratings would satisfy the restrictions implied by the interchangeable model (i.e., invariant loadings and intercepts as shown in Figures 4A,B). Note that from a measurement theoretical point of view, mother, father, and teacher ratings would typically not be seen as interchangeable methods, because they are not sampled from the same “universe” of methods; here, we use these data merely to illustrate MI analyses in the interchangeable MTMM model and do not imply that the ratings should be treated as interchangeable.

The fit statistics are presented in Table 4. Parameter estimates for the final models are given in Table 5. We found that a model with invariant loadings and intercepts for all three types of raters (mother, father, and teacher) was not tenable for either HI or IN, even if the less restrictive version of the model with indicator-specific traits was used. One reason was that in this model, the latent means are implicitly assumed to be equal across all interchangeable methods—this assumption was already rejected in our initial analyses of the simple CFA model.

**Table 4:** **Goodness of fit statistics for different versions of the CFA-MTMM model for interchangeable methods fit to the HI and IN multirater data set**.

**Model**	**χ^2^**	***df***	***p***	***RMSEA***	***CFI***	***TLI***	***SRMR***	**χ^2^Δ**	***df*Δ**	***p*(χ^2^Δ)**	***AIC***
**HYPERACTIVITY/IMPULSIVITY**											
All three raters; equal loadings and intercepts	378.01	31	<0.001	0.13	0.948	0.939	0.16				9867
Mothers and fathers only; equal loadings and intercepts	7.34	8	0.50	0.00	1.000	1.000	0.01				6102
Mothers and fathers only; equal loadings, intercepts, and residual variances	**12.70**	**11**	**0.31**	**0.02**	**1.000**	**0.999**	**0.01**	**5.36**	**3**	**0.15**	**6102**
Mothers and fathers only; equal loadings, intercepts, residual variances, and method factor variances	16.81	12	0.16	0.03	0.999	0.999	0.03	4.11	1	0.04	6104
**INATTENTION**											
All three raters; equal loadings and intercepts	381.16	31	<0.001	0.13	0.949	0.941	0.09				9556
Mothers and fathers only; equal loadings and intercepts	9.42	8	0.31	0.02	1.000	0.999	0.01				5648
Mothers and fathers only; equal loadings, intercepts, and residual variances	10.10	11	0.52	0.00	1.00	1.00	0.01	0.68	3	0.88	5643
Mothers and fathers only; equal loadings, intercepts, residual variances, and method factor variances	**11.15**	**12**	**0.52**	**0.00**	**1.000**	**1.000**	**0.02**	**1.05**	**1**	**0.31**	**5642**

Note: In order to save space, we only present results for the model version with indicator-specific traits (Figure 4B), given that the model version with a single trait (Figure 4A) did not fit well for any rater combination. RMSEA, root mean square error of approximation; CFI, comparative fit index; TLI, Tucker-Lewis index; SRMR, standardized root mean square residual; AIC, Akaike's information criterion. For both constructs, the initial model included all three rater types. Subsequent models included only mother and father ratings (dropping teacher ratings from the analysis). Bold-face indicates best-fitting models.

**Table 5:** **Parameter estimates in the CFA-MTMM models for interchangeable methods fit to mother and father ratings of HI and IN**.

**Parameter label**	**Hyperactivity/impulsivity (*j* = 1)**			**Inattention (*j* = 2)**		
	**Estimate**	***SE***	**Standardized estimate**	**Estimate**	***SE***	**Standardized estimate**
**TRAIT FACTOR LOADINGS**						
λ_1*j*_	1.00^a^	—	0.84; 0.89^b^	1.00^a^	—	0.84
λ_2*j*_	1.00^a^	—	0.88; 0.91^b^	1.00^a^	—	0.87
λ_3*j*_	1.00^a^	—	0.84; 0.88^b^	1.00^a^	—	0.85
**METHOD FACTOR LOADINGS**						
γ_1*j*_	1.00^a^	—	0.37	1.00^a^	—	0.47
γ_2*j*_	0.88	0.05	0.34	1.05	0.05	0.41
γ_3*j*_	0.99	0.05	0.07	0.98	0.05	0.43
**ERROR VARIANCES**						
*Var*(ε_1*j*_)	0.09	0.01	0.08; 0.08^b, c^	0.07	0.01	0.08^c^
*Var*(ε_2*j*_)	0.09	0.01	0.07; 0.08^b, c^	0.09	0.01	0.08^c^
*Var*(ε_3*j*_)	0.09	0.01	0.08; 0.09^b, c^	0.09	0.01	0.10^c^
**LATENT MEANS**						
*E*(*T*_1*j*_)	1.09	0.04		0.98	0.03	
*E*(*T*_2*j*_)	0.95	0.04		1.17	0.04	
*E*(*T*_3*j*_)	1.08	0.04		1.09	0.04	
**LATENT VARIANCES**						
*Var*(*T*_1*j*_)	0.86	0.06		0.58	0.04	
*Var*(*T*_2*j*_)	0.92	0.06		0.88	0.06	
*Var*(*T*_3*j*_)	0.78	0.06		0.66	0.05	
*Var*(*M*_*j*1_)	0.26	0.04		0.18^d^	0.02	
*Var*(*M*_*j*2_)	0.14	0.03		0.18^d^	0.02	

*For hyperactivity/impulsivity, a model of strong invariance for all raters and equal means across mother and father ratings was chosen. For inattention, a model of strict invariance for mother and father ratings was chosen. λ_*ijk*_, trait factor loading (i, indicator; j, trait; k, method/rater); γ_*ijk*_, indicator-specific factor loading; α_*ijk*_, intercept; Var(ε_*ijk*_), error variance. The methods used here are mother report (k = 1), father report (k = 2), and teacher report (k = 3)*.

a *Parameter fixed for identification*.

b *Standardized loadings and standardized error variances for HI differed between raters for the same variable, because the method factor variances were allowed to differ in the final model. The standardized loadings and error variances are therefore given separately for each rater type in the following order: (1) mothers, (2) fathers*.

c *Standardized residual variances indicate 1 – R^2^ and can be interpreted as coefficients of unreliability [(1 – Rel(Y_ijk_)] for each variable*.

d *Method factor variances were set equal across mother and father reports in this model*.

*Dashes indicate fixed parameters for which no standard errors are computed*.

In contrast, an indicator-specific trait model for mother and father reports only (dropping teacher reports from the analysis) fit both the HI and IN data well, showing that mother and father ratings satisfied the conditions of interchangeability implied by the model in this application. This parallels our findings from the previous analyses according to which mother and father ratings were more similar to one another than they were compared to teacher ratings. For both HI and IN, mothers and fathers shared the same or very similar means as indicated by previous analyses. (The baseline model for IN yielded a significant mean difference between mother and father reports; this was likely because there was more statistical power to detect mean differences in the combined model with all three raters. Nonetheless, the mean difference between mothers and fathers was very small also in the initial analysis).

We also tested more strict models of interchangeability for mother and father ratings, in which we also constrained (a) the error variances and (b) the method factor variances to be equal across mother and father ratings. Chi-square difference tests revealed that for HI, equal error variances were tenable, but not equal method factor variances. In contrast, for IN, both the assumption of equal error variances and the assumption of equal method factor variances were acceptable according to the chi-square difference test. In summary, mother and father ratings of IN could be viewed as strictly interchangeable in the sense of the model, whereas for HI the ratings could be viewed as interchangeable except for the amount of method factor variance.

## Discussion

Researchers frequently use different raters as methods in MTMM studies. Often, ratings are provided on comparable items or scales. In these cases, one can examine whether (1) different raters use the items or scales in equivalent ways (i.e., whether MI holds across methods) and (2) whether there is convergent validity of latent mean levels across methods. This opens up new possibilities for studying method effects in more detail. Whereas traditional approaches to MTMM analyses (such as Campbell and Fiske's 1959; MTMM matrix or conventional CFA-MTMM models) have typically focused exclusively on (observed or latent) relationships (correlations) between *different* TMUs, the MI approach presented here first of all examines the relationships between indicators and latent variables *within* each TMU. In this article, we proposed to analyze MI using a baseline MTMM model without method factors in the first step of the analysis. Using this model, researchers can first of all test whether the proposed factor structure holds within corresponding TMUs and second, whether the way indicators relate to latent factors within a TMU is comparable across methods. This allows researchers to examine whether supposedly equivalent concepts that are measured by the same indicators (but different methods) have similar relationships with their indicators for different methods. (This may be termed an examination of the “convergent validity of measurement properties.”). If they do, this may increase a researcher's confidence that similar concepts are indeed measured by each method or at least that the indicators “function” similarly across methods. If they don't, then a researcher may question whether the concepts can be seen as equivalent across methods, warranting further study of what exactly is measured by each method and in which ways concepts or item meanings might differ across methods.

Non-invariant intercepts or loadings across methods can indicate that the scales have a different meaning for different methods. For example, a certain behavior may be less relevant for the definition of a construct for one type of rater than for another. Consider, for instance, symptoms of ADHD. A specific ADHD-IN symptom may be highly relevant for parents' view of their children, but not so critical for teachers' view (maybe because it does not occur in the school context), thus leading to different factor loadings or intercepts for the same symptom. The finding of measurement non-invariance can thus shed more light on how different indicators (e.g., ADHD symptoms) “function” for different types of raters.

It is interesting to note that our approach of beginning an MTMM analysis with a thorough investigation of the measurement properties within and across TMUs seems to be well in line with Fiske and Campbell's (1992) later view of the original Campbell and Fiske (1959) MTMM approach. In their 1992 review, Fiske and Campbell proposed to “settle for the practice of studying ‘TMUs’,” given that “method and trait or content are highly interactive and interdependent” (p. 394). Examining MI across TMUs is one component of such an analysis strategy that places more emphasis on what is measured within each TMU rather than just on correlations between TMUs.

While traditional MTMM analyses focus exclusively on correlation or covariance structures (Campbell and Fiske, 1959), we propose to routinely consider means in the analyses as well, which is a novel aspect in MTMM research. By including means in the analysis, MI across methods can be more fully tested and, if strong MI can be established, latent means can be compared across methods to examine the degree of convergent validity of mean levels across methods. When using just the covariance matrix (and no means), researchers can test for loading (weak factor) invariance and invariant error variances, but not for intercept (strong) MI. In our empirical example, we found that the intercepts were non-invariant across some methods for one of the constructs, indicating differences in scale difficulty across methods. This information could not have been obtained without including means in the analysis.

In addition, when only covariances or correlations are analyzed, latent means cannot be compared across methods. Strong MI is a prerequisite for interpreting latent mean differences across methods in a straightforward way. With non-invariant intercepts and/or loadings, latent mean differences across methods may be difficult to interpret, because the measurements would not be in the same metric in this case. In cases of non-invariance, mean differences would represent a mixture between rater biases and measurement bias (differences in scale use). This was the case for parent vs. teacher ratings of IN in our empirical example. Of course, the question of interpretability depends on the particular substantive application and is also a matter of degree rather than “all or nothing.” For example, if violations of MI are small, approximate MI (van de Schoot et al., 2013) may still hold, warranting a proper interpretation of latent mean differences across methods.

If strong MI can be established across methods (such as in our empirical application to the HI construct), then a researcher can meaningfully test whether methods converge in the assessment of the latent mean level of a construct in a given population. In our HI example, this was the case for mothers vs. fathers, but not for parents vs. teachers. This showed that there was a lack of convergent validity of mean levels across methods (or a true difference between the home and school contexts), even though the convergent validity in terms of the correlations between parents and teachers was quite strong. This issue is especially critical when researchers want to draw conclusions about, for example, the overall level of a problem such as HI. In this case, they would come to different conclusions based on parent vs. teacher ratings in the present example. It is therefore important to examine the convergent validity of mean levels across raters in such cases.

Of course, testing for MI and comparing latent means only makes sense when methods used comparable measurement instruments (items and response scales) to begin with. When very different methods are used (e.g., ratings vs. physiological measures of stress), tests of MI are typically not meaningful, especially when scoring procedures differ between methods (e.g., 4-point Likert scale vs. cortisol concentration in nmol/L). When different methods used similar response scales as in the examples presented in this paper, but strong MI cannot be established, observed mean differences for corresponding indicators across methods still provide valuable information, as they indicate method effects at the measurement level (i.e., differences in scale use; see discussion above).

### Different models

In this article, we demonstrated that including mean structures and testing for MI is not only an issue of potential substantive interest in MTMM analyses. With respect to more complex CFA-MTMM models with method factors, we showed that MI is relevant to these models especially for two reasons: (1) the definition of trait and method factors may require strong MI for a meaningful interpretation of structural parameters such as latent trait and method factor means and variances as well as individual scores on these latent variables or (2) at least strong MI is implied by a CFA model for interchangeable methods. Therefore, MI is not just something that researchers may or may not be interested in when analyzing MTMM data; instead, depending on the model, MI can be a prerequisite for the proper interpretation of one's MTMM model or for conclusions about whether methods can be seen as interchangeable or not.

We showed that among the more complex models for structurally different methods discussed here, the CT-C(*M* – 1) model makes the least restrictive assumptions in terms of MI. That is, for calculating coefficients of convergent validity (consistency) and method specificity in this model or for the interpretation of trait and method factors in general, MI beyond configural invariance is not required. The only case in which MI can become relevant in the CT-C(*M* – 1) model is when researchers want to interpret unstandardized structural regression coefficients or latent mean differences derived from these coefficients.

In contrast, the latent difference and latent means models require MI across methods by definition. When different methods used comparable items that were measured on the same scale (or rescaled to the same metric) and provided that strong MI across methods can be established, then the latent difference and latent means models provide a meaningful and straightforward estimation of mean method effects, because means can be directly estimated for the method factors in these models. In contrast, in the CT-C(*M* – 1) model, mean method effects are not directly estimated in terms of method factor means, because the method factors in this model have means of zero by definition. Nonetheless, mean method effects can also be analyzed in the CT-C(*M* – 1) model as shown in detail in Geiser et al. (2012). An advantage of the CT-C(*M* – 1) model is that it can be used even when different methods used completely different metrics (e.g., self-reports on a 4-point Likert scale vs. cortisol concentrations as measures of stress) or when MI does not hold.

The latent difference model is less restrictive with regard to MI than the latent means model, as the latent difference model can still be used in cases of partial MI (when at least one non-reference method shows strong MI relative to the reference method). In contrast, a proper interpretation of the common trait in the latent means model requires that strong MI between all methods be established. Despite these differences between the CT-C(*M* – 1), latent difference, and latent means models, all three models provide meaningful definitions of trait and method factors. The choice of a particular model depends in part on a researcher's specific goals in a given application.

Another area of MTMM research for which MI plays a role is when researchers study interchangeable methods. We considered Eid et al.'s (2008) CFA-MTMM model for interchangeable methods separately, because it is designed for a different data structure (interchangeable methods) than the CT-C(*M* – 1), latent difference, and latent means models (which are designed for structurally different methods). If, for example, a researcher wants to test whether methods that, based on theory, are conceived of as interchangeable truly are interchangeable in a statistical sense, he or she should use an appropriate CFA-MTMM model for interchangeable methods and test for MI. If this assumption is rejected, the methods in question may be better viewed as structurally different. In this case, one of the models for structurally different methods [CT-C(*M* – 1), latent difference, or latent means] may be more appropriate.

## Conclusion

Most MTMM studies so far have focused on assessing convergent validity in terms of correlations between methods or raters selected to measure the same constructs. We argued that useful incremental information about method effects can be gained from including mean structures in MTMM models and testing for MI across methods. Furthermore, we showed that MI is relevant in more complex CFA-MTMM models with method factors, either because the definitions of trait and method factors imply MI for a meaningful interpretation of structural parameters or because the type of method (interchangeable vs. structurally different) may or may not imply MI across methods. We hope that researchers will find our article useful as a guide for future, more fine-grained studies of method effects.

### Conflict of interest statement

The authors declare that the research was conducted in the absence of any commercial or financial relationships that could be construed as a potential conflict of interest.

## Footnotes

^1^Latent correlations are less biased than observed correlations only if correlated errors of measurement do not exist or if such correlated errors are properly modeled.

^2^In addition to full invariance that requires all corresponding parameters to be equal across raters, models of partial invariance have also been discussed in the general MI literature. Partial MI means that invariance is tenable only for a subset, but not all indicators (e.g., Byrne et al., 1989). In addition, in the MTMM case, measurement parameters may be invariant across some, but not all methods as shown in the empirical example section.

^3^Some authors have recommended that strict invariance be established before latent mean comparisons are conducted (see, e.g., Wu et al., 2007). Strict invariance is only necessary, however, when correlated errors of measurement exist and are not properly modeled. We recommend that researchers pay careful attention to tests of model fit to detect potential error correlations and that such correlations—if they exist—be properly modeled with additional latent variables.

^4^ The CT-C(M – 1) model version is slightly more restricted than Eid et al.'s (2003) original model version. We chose to present the more restricted version here, given its direct correspondence to and mathematical equivalence with the latent difference and latent means models. The differences between Eid et al.'s (2003) model and the version presented here are explained in Geiser et al. (2012); the general MI issues discussed below apply to either version of the model.

^5^Indicator-specific loadings γ_*ijk*_ were not invariant across methods in the present application, indicating that different methods reflected indicator-specific effects differently. In particular, as can be seen from Table 2 teacher ratings showed much weaker (and partly insignificant) loadings on the *IS_ij_* factors compared to parents, indicating that teachers did not differentiate as much between different facets of ADHD as did parents. This could potentially be explained by Halo effects that may have been more significant for teacher as compared to parent ratings in the present application.
