# Supplementary material for: Testing for measurement invariance and latent mean differences across methods: interesting incremental information from multitrait-multimethod studies
Source: Front Psychol. 2014 Oct 30;5:1216. doi: 10.3389/fpsyg.2014.01216 (PMC4214357; doi:10.3389/fpsyg.2014.01216)
Supplement: Supplementary file 1 [file DataSheet1.DOCX]

**Appendix A**

**Formal Definition of Indicator-Specific Factors in the Model Shown in Figure 2**

The starting point for the definition of the indicator-specific factors *IS_ij_* in the model in Figure 2 is the following latent regression analysis:

*E*(*T_ijk_* | *T*_1_*_jk_*) = α*_ijk_* + λ*_ijk_T*_1_*_jk_*, (A1)

where *i* ≠ 1, α*_ijk_* and λ*_ijk_* are real constants, and *E*(*T_ijk_* | *T*_1_*_jk_*) is the conditional expectation (regression) of *T_ijk_* given the reference true score variable *T*_1_*_jk_* (*i* = 1).The residuals of this latent regression analysis are called indicator-specific variables *IS_ijk_* and are defined as follows:

*IS_ijk_* := *T_ijk_* – *E*(*T_ijk_* | *T*_1_*_jk_*), (A2)

where the “:=” symbol indicates a definition.

It is assumed that the indicator-specific variables *IS_ijk_* measure a common (i.e., method-unspecific) indicator-specific factor *IS_ij_* (assumption of homogeneous indicator-specific effects across methods):

*IS_ij_* = γ*_ijk_IS_ijk_*, (A3)

where γ*_ijk_* is a real constant. There is no additive constant (intercept) in Equation A3, because the *IS_ijk_* variables are defined as regression residuals and thus have a mean of zero by definition (e.g., Geiser et al., 2008). In order to establish measurement invariance of the substantive factors *T*_1_*_jk_* across methods, only the intercepts α*_ijk_* and reference factor loadings λ*_ijk_* have to be tested for invariance across methods. This is because the indicator-specific factor loadings γ*_ijk_* are not relevant for the measurement of the substantive factors *T*_1_*_jk_*.

**Appendix B**

**Mplus MODEL Statements for the Specification of MTMM Measurement Models**

**CFA Model (Figure 1) For HI**

This model is a baseline model of configural invariance that involves no parameter equality constraints across methods.

model:

! Mother report HI

HIMOM by HI1momT3@1 HI2momT3 HI3momT3;

! Father report HI

HIDAD by HI1dadT3@1 HI2dadT3 HI3dadT3;

! Teacher report HI

HITEA by HI1taT3@1 HI2taT3 HI3taT3;

**CFA Model With Indicator-Specific Factors (Figure 2) For HI**

This a baseline model of configural invariance that involves no parameter equality constraints across methods.

model:

! Mother report HI

HIMOM by HI1momT3@1 HI2momT3 HI3momT3;

! Father report HI

HIDAD by HI1dadT3@1 HI2dadT3 HI3dadT3;

! Teacher report HI

HITEA by HI1taT3@1 HI2taT3 HI3taT3;

! I - 1 Indicator-specific factors to model parcel-specific effects

! The first parcel serves as reference

IS2 by HI2momT3@1 HI2dadT3 HI2taT3;

IS3 by HI3momT3@1 HI3dadT3 HI3taT3;

! Indicator-specific factors are not allowed to correlate with reference factors

HIMOM HIDAD HITEA with IS2@0 IS3@0;

**CFA Model With Indicator-Specific Factors (Figure 2) For HI With Invariant Parameters**

This model involves invariant loadings and intercepts across methods as well as equal means across mother and father reports.

model:

! Mother report HI

HIMOM by HI1momT3@1

HI2momT3 (lambda2)

HI3momT3 (lambda3);

! Father report HI

HIDAD by HI1dadT3@1

HI2dadT3 (lambda2)

HI3dadT3 (lambda3);

! Teacher report HI

HITEA by HI1taT3@1

HI2taT3 (lambda2)

HI3taT3 (lambda3);

! Mean structure and intercepts

! Set intercepts of reference indicators to zero

[HI1momT3@0 HI1dadT3@0 HI1taT3@0];

! Set remaining intercepts equal across raters

[HI2momT3 HI2dadT3 HI2taT3] (alpha2);

[HI3momT3 HI3dadT3 HI3taT3] (alpha3);

! Estimate latent means

! Set them equal for mother and father reports, but not teacher reports

[HIMOM* HIDAD*] (mean); [HITEA*];

! I - 1 Indicator-specific factors to model parcel-specific effects

! The first parcel serves as reference

IS2 by HI2momT3@1 HI2dadT3 HI2taT3;

IS3 by HI3momT3@1 HI3dadT3 HI3taT3;

! Indicator-specific factors are not allowed to correlate with reference factors

HIMOM HIDAD HITEA with IS2@0 IS3@0;

**CT-C(M – 1) Model (Figure 3A) For HI With Invariant Parameters**

This model uses mother report as reference method.

model:

! Mother report HI

HIMOM by HI1momT3@1

HI2momT3 (lambda2)

HI3momT3 (lambda3);

! Father report HI

HIDAD by HI1dadT3@1

HI2dadT3 (lambda2)

HI3dadT3 (lambda3);

! Teacher report HI

HITEA by HI1taT3@1

HI2taT3 (lambda2)

HI3taT3 (lambda3);

! Mean structure and intercepts

! Set intercepts of reference indicators to zero

[HI1momT3@0 HI1dadT3@0 HI1taT3@0];

! Set remaining intercepts equal across raters

[HI2momT3 HI2dadT3 HI2taT3] (alpha2);

[HI3momT3 HI3dadT3 HI3taT3] (alpha3);

! Latent regression on mother report reference factor

HIDAD HITEA on HIMOM*;

! Estimate the latent mean of the reference method (mother report)

! and intercepts for the remaining methods

[HIMOM* HIDAD* HITEA*];

! Allow correlation between father and teacher method (residual) factors

HIDAD with HITEA*;

! I - 1 Indicator-specific factors to model parcel-specific effects

! The first parcel serves as reference

IS2 by HI2momT3@1 HI2dadT3 HI2taT3;

IS3 by HI3momT3@1 HI3dadT3 HI3taT3;

! Indicator-specific factors are not allowed to correlate with other factors

HIMOM HIDAD HITEA with IS2@0 IS3@0;

**Latent Difference Model (Figure 3B) For HI With Invariant Parameters**

This model uses mother report as reference method.

model:

! Mother report HI

HIMOM by HI1momT3@1

HI2momT3 (lambda2)

HI3momT3 (lambda3);

! Father report HI

HIDAD by HI1dadT3@1

HI2dadT3 (lambda2)

HI3dadT3 (lambda3);

! Teacher report HI

HITEA by HI1taT3@1

HI2taT3 (lambda2)

HI3taT3 (lambda3);

! Mean structure and intercepts

! Set intercepts of reference indicators to zero

[HI1momT3@0 HI1dadT3@0 HI1taT3@0];

! Set remaining intercepts equal across raters

[HI2momT3 HI2dadT3 HI2taT3] (alpha2);

[HI3momT3 HI3dadT3 HI3taT3] (alpha3);

! Introduce latent difference method factors as phantom variables

! Father report

DDIFF by HI1taT3@0;

HIDAD on HIMOM@1 DDIFF@1;

! Set residual variance to zero

HIDAD@0;

! Teacher report

TDIFF by HI1taT3@0;

HITEA on HIMOM@1 TDIFF@1;

! Set residual variance to zero

HITEA@0;

! Set residual covariances to zero

HIDAD with HITEA@0;

! Estimate the latent mean of the reference method (mother report)

! and the means of the latent difference method factors

[HIMOM* DDIFF* TDIFF*];

! I - 1 Indicator-specific factors to model parcel-specific effects

! The first parcel serves as reference

IS2 by HI2momT3@1 HI2dadT3 HI2taT3;

IS3 by HI3momT3@1 HI3dadT3 HI3taT3;

! Indicator-specific factors are not allowed to correlate with other factors

HIMOM HIDAD HITEA DDIFF TDIFF with IS2@0 IS3@0;

**Latent Means Model (Figure 3C) For HI With Invariant Parameters**

model:

! Mother report HI

HIMOM by HI1momT3@1

HI2momT3 (lambda2)

HI3momT3 (lambda3);

! Father report HI

HIDAD by HI1dadT3@1

HI2dadT3 (lambda2)

HI3dadT3 (lambda3);

! Teacher report HI

HITEA by HI1taT3@1

HI2taT3 (lambda2)

HI3taT3 (lambda3);

! Mean structure and intercepts

! Set intercepts of reference indicators to zero

[HI1momT3@0 HI1dadT3@0 HI1taT3@0];

! Set remaining intercepts equal across raters

[HI2momT3 HI2dadT3 HI2taT3] (alpha2);

[HI3momT3 HI3dadT3 HI3taT3] (alpha3);

! Introduce latent means factor

! This factor represents the average of the rater factors

T1 by HIMOM@1 HIDAD@1 HITEA@1;

! Introduce method factors

! These factors represent deviations from the average trait factor

! Arbitrarily, the method factor for mothers is dropped,

! because it is redundant

M12 by HIMOM@-1 HIDAD@1;

M13 by HIMOM@-1 HITEA@1;

! Set residual variances to zero

HIMOM@0 HIDAD@0 HITEA@0;

! Set residual covariances to zero

HIMOM with HIDAD@0 HITEA@0;

HIDAD with HITEA@0;

! Estimate the latent means of the trait and method factors

[T1* M12* M13*];

! I - 1 Indicator-specific factors to model parcel-specific effects

! The first parcel serves as reference

IS2 by HI2momT3@1 HI2dadT3 HI2taT3;

IS3 by HI3momT3@1 HI3dadT3 HI3taT3;

! Indicator-specific factors are not allowed to correlate with other factors

HIMOM HIDAD HITEA T1 M12 M13 with IS2@0 IS3@0;

**CFA-MTMM Model for Interchangeable Methods with a General Trait Factor (Figure 3A)**

The example here shows the model for IN in which all loadings, intercepts, residual variances, and method factor variances were set equal across mother and father reports.

! General trait factor with loadings set equal across raters

T1 by IN1momT3@1

IN2momT3 (lambda21)

IN3momT3 (lambda31)

IN1dadT3@1

IN2dadT3 (lambda21)

IN3dadT3 (lambda31)

! Method factor mother reports

M11 by IN1momT3@1

IN2momT3 (gamma21)

IN3momT3 (gamma31);

! Method factor father reports

M12 by IN1dadT3@1

IN2dadT3 (gamma21)

IN3dadT3 (gamma31);

! Traits and methods are uncorrelated

T1 with M11-M12@0;

M11 with M12@0;

! Intercepts are set equal across raters

! Reference indicators (intercepts set to zero to identify the latent trait mean)

[IN1momT3@0 IN1dadT3@0];

! Set remaining intercepts equal across raters

[IN2momT3 IN2dadT3] (alpha21);

[IN3momT3 IN3dadT3] (alpha31);

! Set residual variances equal across raters

IN1momT3 IN1dadT3 (epsilon1);

IN2momT3 IN2dadT3 (epsilon2);

IN3momT3 IN3dadT3 (epsilon3);

! Set method factor variances equal across raters

M11 M12 (methvar);

! Estimate latent trait mean

[T1*];

**CFA-MTMM Model for Interchangeable Methods with Indicator-Specific Trait Factors (Figure 3B)**

The example here shows the model for IN in which all loadings, intercepts, residual variances, and method factor variances were set equal across mother and father reports.

Model:

! Indicator-specific trait factors with loadings set equal across raters

T11 by IN1momT3@1 IN1dadT3@1;

T21 by IN2momT3@1 IN2dadT3@1;

T31 by IN3momT3@1 IN3dadT3@1;

! Method factor mother reports

M11 by IN1momT3@1

IN2momT3 (gamma21)

IN3momT3 (gamma31);

! Method factor father reports

M12 by IN1dadT3@1

IN2dadT3 (gamma21)

IN3dadT3 (gamma31);

! Traits and methods are uncorrelated

T11-T31 with M11-M12@0;

M11 with M12@0;

! Intercepts are set equal across raters

[IN1momT3@0 IN1dadT3@0

IN2momT3@0 IN2dadT3@0

IN3momT3@0 IN3dadT3@0];

! Set residual variances equal across raters

IN1momT3 IN1dadT3 (epsilon1);

IN2momT3 IN2dadT3 (epsilon2);

IN3momT3 IN3dadT3 (epsilon3);

! Set method factor variances equal across raters

M11 M12 (methvar);

! Estimate latent trait means

[T11-T31*];
